# Exploratory analysis of cell-free miRNA expression changes in patients with isolated first seizure and new-onset epilepsy

**DOI:** 10.3389/fmolb.2026.1788160

**Published:** 2026-10-30

**Authors:** Ryan C. Meng, Alexandra Lesko, Fred Robinson, Elizabeth Baraban, Kai H. Darke, Brian D. Piening, Evan J. Fertig

**Affiliations:** 1 Earle A. Chiles Research Institute, Providence Cancer Institute, Portland, OR, United States; 2 Providence Neuroscience Institute, Providence Saint Vincent, Portland, OR, United States; 3 Department of Neurology, Providence Saint Vincent, Portland, OR, United States

**Keywords:** bioinformatics, biomarker, epileptogenesis, miRNA expression, Neurology, new-onset epilepsy, seizure

## Abstract

One in ten individuals will experience a seizure in their lives, but only 43%–61% will be diagnosed with new-onset epilepsy (NOE). Existing methods to distinguish between a first unprovoked seizure and a diagnosis of epilepsy are inadequate and often lead to unnecessary treatment with antiseizure medications. To uncover potential targets associated with NOE, we analyzed cell-free circulating miRNA expression in plasma samples obtained from 20 patients who presented with a first unprovoked seizure. Patients were monitored for 2 years following their initial seizure, and a total of 7 patients experienced a second seizure within the follow-up period. Analysis of the miRNA expression levels revealed 15 differentially expressed miRNAs between patients who experienced only a first unprovoked seizure versus those who had a second seizure. These 15 miRNAs were experimentally linked as regulators to 160 genes related to epilepsy, including genes involved in the mTOR and WNT signaling pathways. These findings highlight a number of key circulating miRNA targets that may be further developed as a biomarker signature in larger cohorts and may offer insights into factors associated with epileptogenesis.

## 1 Introduction

One in ten individuals will experience a first seizure at some point in their lives. Evidence-based guidelines from the American Academy of Neurology and the American Epilepsy Society state that adults who experience a first unprovoked seizure (FUS) have a 43%–61% risk of seizure recurrence within 2 years, thus meeting the criteria for a diagnosis of new-onset epilepsy (NOE) (Krumholz et al., 2015; Fisher et al., 2014). An epilepsy diagnosis is associated with psychological and social consequences such as loss of driving privileges and employment, injury, and mortality. Despite these consequences, antiseizure medications (ASM) reduces the short-term risk of a second seizure in appropriately identified patients (Kim et al., 2006). However, unnecessary ASM use is associated with adverse effects, increased cost, and decreased quality of life (Benbadis, 2010). Thus, identifying individuals with FUS at high risk for NOE could improve morbidity in patients and reduce overall health costs.

Our ability to predict whether a FUS is the initial presentation of NOE or an isolated first seizure (IFS) is limited. Current methods include clinical history review, (Krumholz et al., 2015) neurological examination, electroencephalography (EEG), and neuroimaging. However, these tools have limited ability to predict which patients will have more seizures after a first event. For example, MRI reveals a potentially epileptogenic lesion in only 23% of patients with new onset seizure (Hakami et al., 2013). EEG is abnormal in ∼50% of patients with new onset seizures (Benbadis, 2010). Further, EEG interpretation is subject to bias and poor inter-rater reliability, which leads to misdiagnosis of epilepsy in ∼25% of cases (Benbadis, 2010; Benbadis and Lin, 2008). In addition, 8% of patients with epilepsy will never demonstrate interictal epileptiform discharges during a routine 30-min EEG study (Binnie and Stefan, 1999; Marsan and Zivin, 1970; Salinsky et al., 1987). EEG has been shown to be of higher diagnostic yield immediately after a first seizure, and extended EEGs may be of additional yield, but these may be inconvenient, costly, or infeasible (Chen et al., 2016; King et al., 1998; Lee et al., 2013; Juarez-Garcia et al., 2006). Further, there is limited data regarding the additive effects or covariance of risk factors for seizure recurrence after first seizure (Kim et al., 2006). Overtreatment is also a concern as ∼40% of patients with an FUS who meet the threshold for treatment defined by the International League Against Epilepsy will never have a second seizure or relapse. For these patients, the daily burden of ASM therapy could be unnecessary.

Molecular diagnostics is one promising strategy for the diagnosis of NOE. While the mechanisms of epileptogenesis in humans remain largely unknown, a leading hypothesis is that cellular reprogramming through epigenetic, noncoding RNA, or other regulatory mechanisms, is a key step in early epileptogenesis (Younus and Reddy, 2017; Chen et al., 2017). These hypotheses are supported by studies in animal models for epilepsy and in humans, where significant changes in global and local methylation (Kobow et al., 2013; Dębski et al., 2016), gene expression (Aronica and Gorter, 2007; Pfisterer et al., 2020; Wang L. et al., 2016), miRNA expression (Alsharafi et al., 2015; Korotkov et al., 2017; Srivastava et al., 2016), protein expression, and post-translational modification were associated with epileptogenesis (do Canto et al., 2020; Liu et al., 2010; Keck et al., 2017). While most prior studies have focused on profiling brain tissue, some have shown that a subset of these analytes can be detected in the peripheral blood (Enright et al., 2018; Banote et al., 2021; Hong-Yu, 2018; Kobow et al., 2024). As such, there is potential that one or more of these analytes could be further developed into a diagnostic biomarker.

Here we performed a study to assess changes in cell-free (CF) miRNA in first seizure patients that either did or did not progress to NOE. Assessing a broad spectrum of miRNA across a cohort of individuals, we observed a series of miRNA targets that were reproducibly up- or downregulated in the blood of patients that progressed to NOE. We performed an in-depth analysis of the known regulated gene targets of these miRNAs, which revealed potential links to the etiology of epileptogenesis. These results represent a potential biomarker discovery strategy for the early identification of NOE.

## 2 Materials and methods

This was a prospective, two center, observational study of patients presenting with a FUS. The primary objective of this study was to identify differentially expressed circulating miRNAs associated with progression to NOE.

### 2.1 Ethics & informed consent

All participants consented for participation in compliance with the common rule and good clinical practice guidelines. The study was approved by the Institutional Review Board (IRB) of Providence (IRB protocol 2018000204). All participants signed consent after reviewing benefits and risks of participation with an epileptologist or study coordinator. Participants did not receive any incentives for participation.

### 2.2 Setting

Patients who presented with a FUS at either Providence Saint Vincent Medical Center or Providence Portland Medical Center between 9 September 2018, and 2 December 2024, were eligible for the study. Because of changes to clinical practice during the covid-19 pandemic, enrollment was paused indefinitely in 2020 and ultimately was not resumed. Patients enrolled before 1 November 2019 were referred to the Providence First Seizure Clinic (PFSC) for evaluation by an epileptologist. All blood work was drawn for labs within Providence Saint Vincent or PFSC. Samples were sent to the Providence genomics lab for analysis. Participants were followed from the time they consented to participate until 2 years after their first seizure.

### 2.3 Participants

All participants were 18 years or older and were determined to have had an unprovoked seizure after an evaluation by an epileptologist. Patients were excluded from the study if they.

- Were determined to have had two or more seizures separated by 24 h ≥ 3 months previously.
- Had a clinical evaluation greater than 60 days from the date of their first, unprovoked seizure.
- Were currently taking ASMs.
- Had a first seizure that was determined to have been acute symptomatic.

All eligible patients that consented for participation completed a blood draw. A total of twenty patients who experienced a FUS provided a 4 mL ethylenediaminetetraacetic acid blood draw tube in clinic. Whole blood samples were transferred to the lab and processed within 24 h of collection using a standardized protocol. Plasma was isolated by centrifugation, aliquoted to minimize freeze-thaw exposure, and stored at −80C. All samples underwent identical pre-analytical handling procedures and experienced no more than one freeze-thaw cycle prior to CF miRNA analysis.

Seven of twenty patients later experienced a second seizure within the 2-year follow-up window of our study and met the definition of NOE.

### 2.4 Variables and data sources

Patient demographic and clinical characteristics, including neurological diagnostic information, were abstracted from the medical record. Data abstraction was validated by an epileptologist. Race and ethnicity in the medical record is patient-reported and collected in routine medical care. Race and ethnicity categories were based on USA census definitions. Genomic data was extracted from blood serum samples provided by study participants.

### 2.5 Profiling cell-free miRNA

Following cohort selection and sample extraction, we performed analysis of CF miRNA profiling. Briefly, CF nucleic acid fractions were isolated by Qiagen spin column according to the manufacturer’s instructions and CF miRNA fractions were barcode-tagged and amplified for NanoString miRNA analysis using the nCounter Human v3 miRNA Panel and analyzed via the NanoString nCounter Digital Analyzer. The NanoString nCounter miRNA assay is a direct digital counting platform and does not rely on reverse transcription or PCR amplification of target miRNAs. Following completion of the run, library quality assurance was conducted. Raw data files in reporter code count format were exported from the nCounter Digital Analyzer for downstream analysis.

### 2.6 Normalization of samples for analysis

After sequencing and quality control assessment, raw miRNA counts were normalized for differential expression analysis. Because no universally stable housekeeping genes have been established for plasma CF miRNA, reference targets were empirically selected based on stability within the study’s cohort rather than assumed *a priori*. Several methods were utilized to determine the most stable targets to use for normalization because there was significant variability among the designated housekeeping genes (Supplementary Figure S1). Normalization methods included normalization to the housekeeping targets designated by NanoString, normalization to the targets determined via the NanoTube R package and using the methods described in Gouin et al. (Class et al., 2023) and RefFinder (Wiley, 2015; Xie et al., 2023). Successful normalization was assessed by relative log expression plots and it was determined that RefFinder provided the most accurate targets for sample normalization (Supplementary Figure S2). Briefly, a raw counts table of miRNA expression for all experimental samples was submitted to RefFinder to generate weights for each miRNA and to generate a consensus list of miRNAs with the lowest geometric weights for selection as reference genes for normalization. Based on the consensus weights assigned by RefFinder, *hsa-miR-299-5p*, *hsa-miR-140-5p*, *hsa-miR-18a-5p*, *hsa-miR-1285-5p*, *hsa-miR-301a-3p*, and *hsa-miR-186-5p* were all selected as the most stable targets for normalization. The selected miRNAs demonstrated minimal variability across all samples and no systematic differences between clinical outcome groups, supporting their biological stability for normalization in this dataset.

### 2.7 Differential miRNA expression analysis

Following normalization, we conducted differential expression analysis of the miRNA targets using the NanoStringDiff R package (Wang H. et al., 2016). Differences in miRNA expression across participants who experienced only a FUS versus those who were later diagnosed with NOE were assessed using a generalized linear model of the negative binomial family to characterize the data. Differential expression results were reported as log2 fold change values as calculated by NanoStringDiff. Individual miRNAs were deemed statistically significant if they possessed a q-value of less than or equal to 0.05 determined by NanoStringDiff. The results of the differential expression analysis were visualized using the ggvolc package in R (Theodosiou, 2025).

### 2.8 Enrichment analysis of differentially expressed miRNAs

The differentially expressed miRNA were then analyzed utilizing mirPathDB 2.0 (Kehl et al., 2020). A list of all miRNAs determined to be differentially expressed between patient subgroups (Supplementary Table S3) were submitted to mirPathDB. The database returned a detailed list of genes predicated, or experimentally proven, to be targeted by our differentially expressed miRNAs. The list of genes that were shown to be targeted by our differentially expressed miRNAs via experimental evidence was compiled from a literature search and downstream analysis. We conducted enrichment analysis utilizing the list of genes shown to be targeted by the identified miRNAs using DAVID (Sherman et al., 2022) and enriched pathways were visualized using clusterProfiler (Yu et al., 2012) and StringDB (Szklarczyk et al., 2023).

## 3 Results

A total of 20 patients met the study criteria, consented to participate, and provided a blood draw (Supplementary Figure S4). The median age of patients who consented and enrolled into the study was 34 years, 35% (7 of 20) were female, and 85% (17 of 20) were White (Table 1). The median turn-around time from first seizure to blood draw was 13 days. Most patients (75%, 15 of 20) exhibited normal EEG results following their first seizure. Patients were monitored for 2 years past the date of their first seizure for an occurrence of an additional seizure. One patient was monitored for less than 2 years (361 days) because they transferred out of our hospital system. A total of 35% (7 of 20) patients experienced an additional seizure within 2 years and met the definition of NOE.

**TABLE 1:** Patient demographic and clinical characteristics.

Characteristic	Total	New onset epilepsy	Isolated first seizure
N (%)	20 (100)	7 (100)	13 (100)
Age at 1st unprovoked seizure, median (IQR)	34 (30.2, 41.2)	31.0 (26.5, 35.5)	35.0 (33.0, 42.0)
Female, n (%)	7 (35)	3 (43)	4 (31)
Hispanic ethnicity, n (%)			
Yes	0	0	0
Chose not to disclose	1 (5)	0	1 (8)
Race, n (%)			
Asian	1 (5)	1 (14)	0
Black	1 (5)	1 (14)	0
White	17 (85)	5 (71)	12 (92)
Chose not to disclose	1 (5)	0	1 (8)
Days between 1st seizure and blood draw, median (IQR)	13 (5.8, 22.8)	12 (8.5, 20.0)	14 (5.0, 19.0)
Results of 1st MRI or CT following 1st seizure, n (%)			
None	16 (80)	6 (86)	10 (77)
Epileptogenic abnormality	1 (5)	0	1 (8)
Non-epileptogenic abnormality	1 (5)	0	1 (8)
MRI not completed	2 (10)	1 (14)	1 (8)
Results of 1st EEG following 1st seizure, n (%)			
None	0	0	0
Normal	15 (75)	4 (57)	11 (85)
Epileptiform abnormality	3 (15)	2 (29)	1 (8)
Non-epileptiform abnormality	2 (10)	1 (14)	1 (8)
Results of neurological exam following 1st seizure, n (%)			
Normal	20 (100)	7 (100)	13 (100)
Abnormal	0	0	0
Patients with a relapse, n (%)	7 (35)	7 (100)	0

^a^ Had one or more unprovoked seizures after first isolated seizure. IQR: interquartile range, MRI: magnetic resonance imaging, CT: computed tomography, EEG: electroencephalogram.

### 3.1 Differential miRNA expression analysis

All miRNA samples were normalized using RefFinder, and differential expression analysis was performed to identify differences between patients who experienced an isolated first seizure (ISF) versus those who had NOE. Fifteen miRNAs had significant differences in expression between patients who had an ISF and those with NOE (q-value <0.05, Figure 1A; Supplementary Table S3). Of the 15 differentially expressed miRNAs, 13 had an increased expression in the cohort of patients with NOE following first seizure and 2 had decreased expression following first seizure.

**FIGURE 1:**
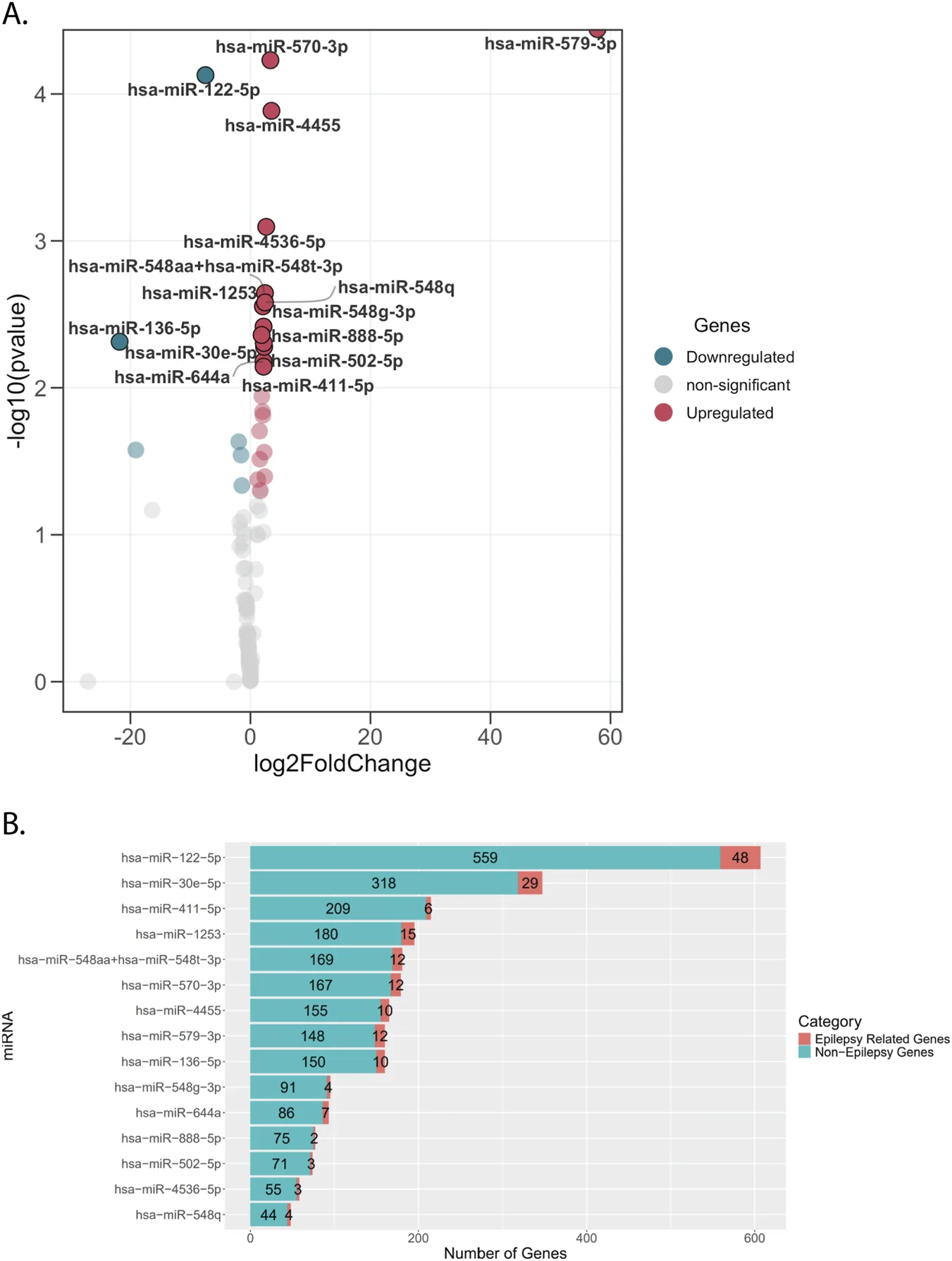
Differential Expression Analysis of miRNA. **(A)** Volcano plot of differentially expressed miRNAs between Isolated Seizure and NOE. Genes in red are upregulated in NOE with respect to Isolated seizure, while genes in blue are downregulated. **(B)** Bar chart of genes shown to be regulated by our 15 differentially expressed miRNAs stratified by epilepsy-related and non-epilepsy related genes.

### 3.2 Database analysis of targeted genes by differentially expressed miRNAs

We next used mirPathDB 2.0 to assess the genes targeted by our 15 differentially expressed miRNAs using only interactions that were shown in the literature to be experimentally linked to epilepsy. We first identified 2,253 unique gene targets based on published experimental evidence. Previous work in the field has identified 974 genes related to epilepsy utilizing multiple databases (Zhang et al., 2024). Utilizing these epilepsy databases, we identified 160 unique genes related to epilepsy targeted by our miRNAs (Figure 1B).

### 3.3 Biological pathway analysis of targeted genes by differentially expressed miRNAs

We next performed pathway enrichment analysis using the Kyoto Encyclopedia of Genes and Genomes (KEGG) (Kanehisa and Goto, 2000) and DAVID functional enrichment. We observed a total of 66 different KEGG pathways that were enriched based on the genes regulated by our differentially expressed miRNAs (Figure 2). Interestingly, most enriched pathways in our dataset were of viral origin. Pathways relevant to epilepsy research enriched in our dataset were the Wnt and mTOR signaling pathways. Utilizing the stringDB program, we generated networks of the genes involved in the Wnt and mTOR signaling pathways to show interactions between the genes (Figures 3A,B). This revealed four different genes previously shown to be related to epilepsy involved in these pathways (*AKT3*, *BRAF*, *PIK3R2*, *TBL1XR1*).

**FIGURE 2:**
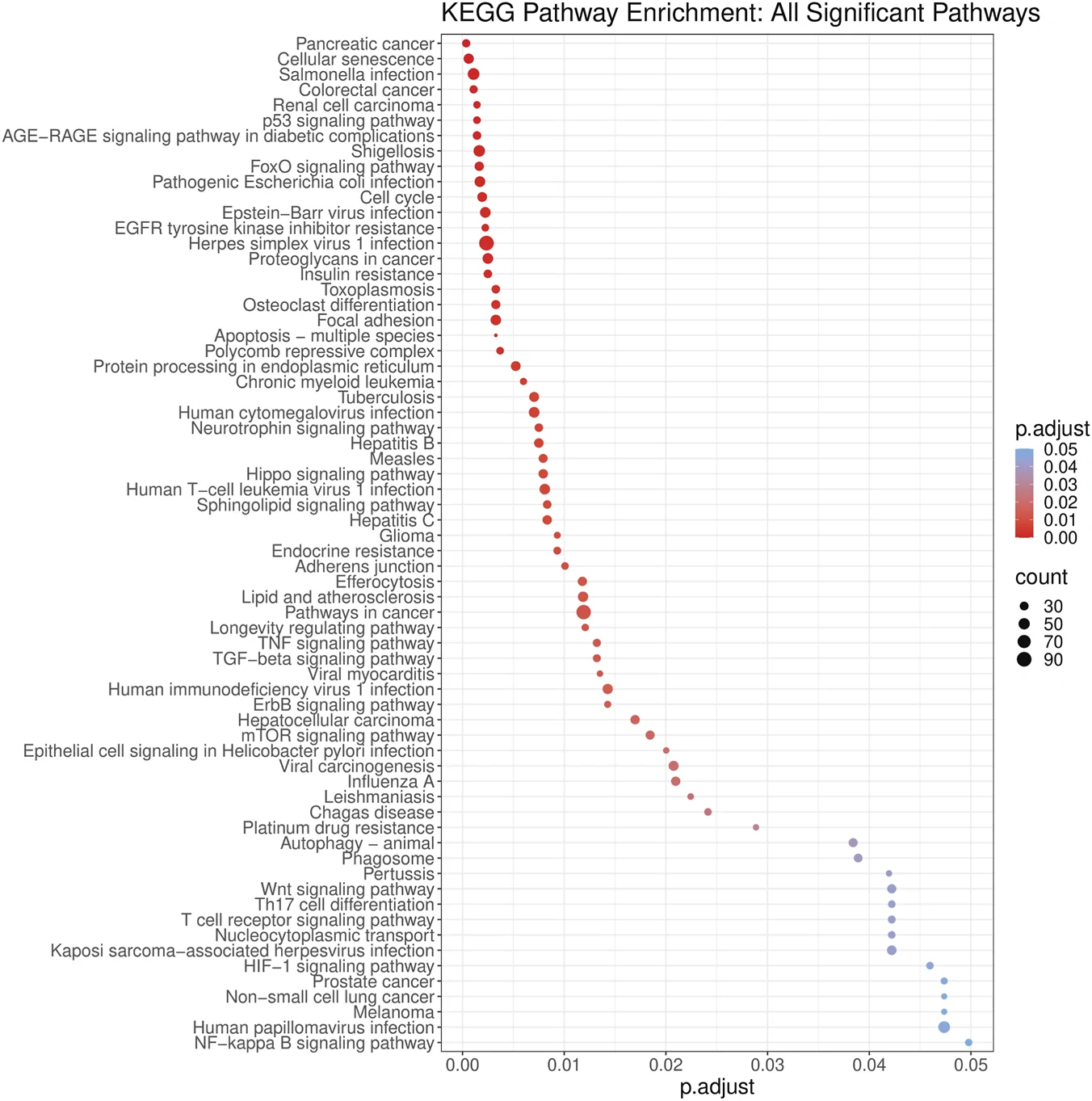
KEGG Pathway Analysis. Dot plot representation of significant KEGG pathways from genes targeted by our miRNAs.

**FIGURE 3:**
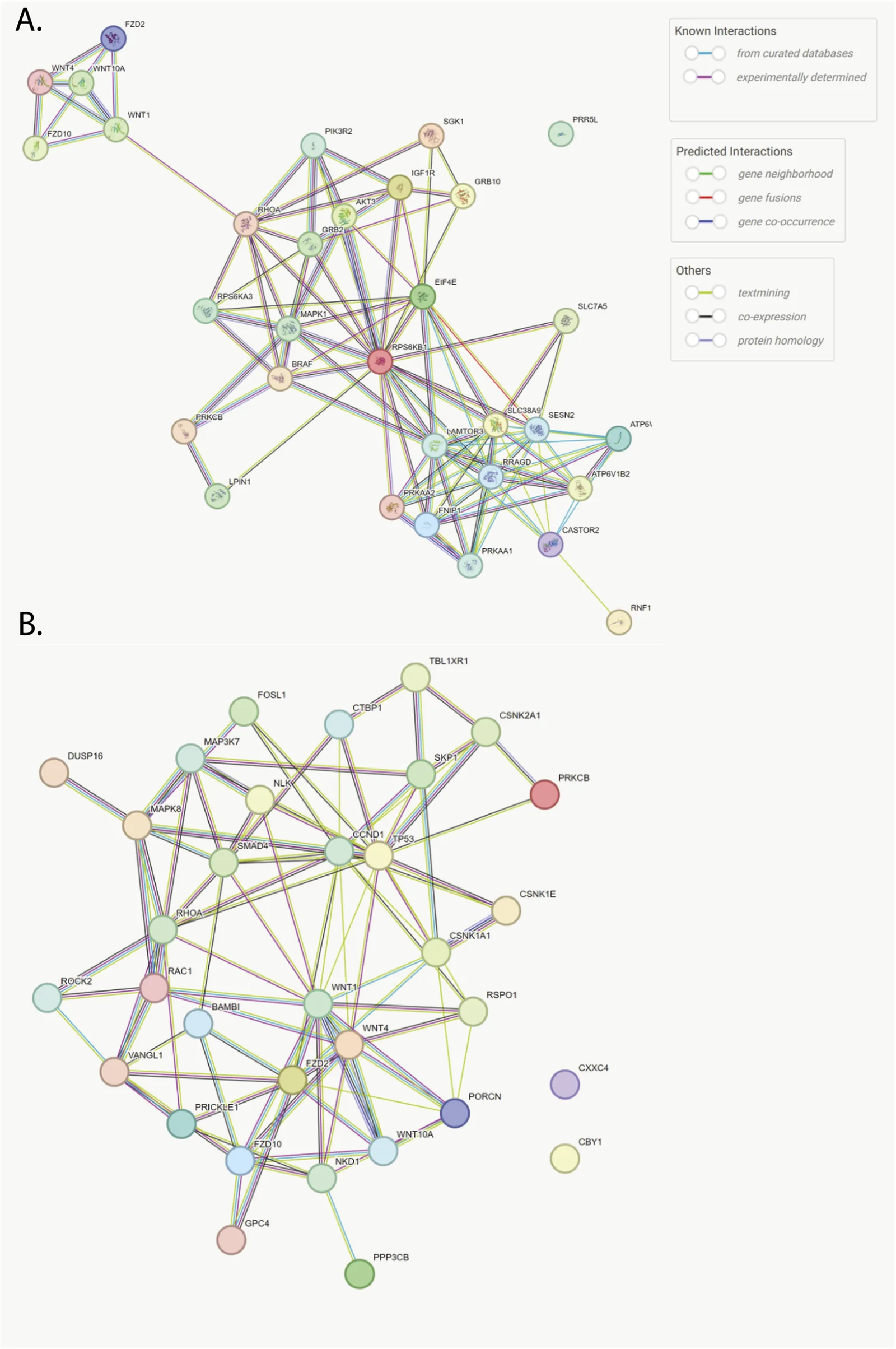
String-DB Diagrams. These diagrams highlight genes targeted by our differentially expressed miRNAs. **(A)** String-DB diagram of the Wnt signaling pathway. **(B)** String-DB diagram of the mTOR signaling pathway.

## 4 Discussion

Our study analyzed the expression of miRNAs in a cohort of 20 patients who had a FUS and, following a 2-year follow-up period, were ultimately diagnosed with either IFS or NOE. We identified 15 differentially expressed miRNAs observed between NOE and IFS patients. These miRNAs were experimentally linked as regulators to 2,253 different genes, with 160 of those genes related to epilepsy, and many more involved in neurological pathways such as Wnt and mTOR signaling. An advantage of our study was that our participants were screened by a group of epilepsy specialists and none of the participants were taking ASMs, which could affect miRNA expression.

Previous studies highlighted the potential for miRNAs as a biomarker to predict epilepsy, and there have been many studies in both human and mouse models that implicated miRNAs in various neurological disorders. For example, upregulation of *hsa-mir-579-3p*, one of the miRNAs identified in this study, has been shown to be a marker for susceptibility to Parkinson’s disease (Yılmaz et al., 2016). Patients diagnosed with epilepsy are at a greater risk of developing Parkinson’s disease (Hwang et al., 2024), and the elevated expression of *hsa-mir-579-3p* in only our NOE patients would corroborate that notion. In contrast, one of the two miRNAs with reduced expression in our NOE cohort was *hsa-mir-122-5p*, which has been shown to exert neuroprotective effects (Yu et al., 2023).

The differentially expressed miRNAs identified here have been shown to regulate over 2200 different genes. With enrichment analysis using DAVID, some of these genes have been implicated in biological pathways related to epileptogenesis such as the mTOR signaling pathway (Zhao et al., 2023). Upregulation of the mTOR pathway has been associated with increased neuronal hyperexcitation, neuroinflammation, and cell death (LaSarge and Danzer, 2014), which is consistent with the enrichment of the apoptosis pathway in our data. mTOR inhibitors are also seen as a potential therapy option for patients with epilepsy. (Galanopoulou, 2012) More recent studies have identified the Wnt signaling pathway as a potential source of neuronal excitability (Rubio et al., 2020), which could make it a target for the treatment of epilepsy. miRNAs play a key role in regulating many genes involved in these epilepsy related pathways and further studies could help elucidate key genes related to epileptogenesis.

Several miRNAs that have previously been identified as potential biomarkers or treatment targets for epilepsy were absent from our list of differentially expressed miRNAs. For example, upregulation of *mir-134* has been linked to temporal lobe epilepsy (TLE) in humans and rodents (Henshall et al., 2016; Ghafouri-Fard et al., 2022) and silencing *mir-134* in murine models of epilepsy has a therapeutic effect (Jimenez-Mateos et al., 2012). Similarly, *mir-132* has been shown to be upregulated in adults with TLE and in murine models of epilepsy (Ghafouri-Fard et al., 2022).

There are several possible reasons why we did not observe these, and other miRNAs associated with epilepsy, in our cohort. The majority of miRNA biomarker studies for epilepsy have been conducted in brain tissues from animal models or patients with confirmed TLE (Ghafouri-Fard et al., 2022). In these populations, ASM use and longer-term epilepsy could both affect miRNA expression. In contrast, our cohort consisted only of patients who had recently experienced a first seizure, were treatment naive, and had no prior history of seizures. As such, our patient population was distinct from those in previously published studies. Since we were specifically looking at expression differences between ISF and NOE in serum, it is not entirely surprising that we observed a different set of differentially expressed miRNAs than previous studies that were focused on brain tissue from patients with TLE. Our sample size might have also limited our ability to replicate previous miRNA studies of TLE. Only 7 of the patients in our sample were ultimately diagnosed with NOE. Relatively few of these patients had TLE and our study was not powered to detect for expression differences among different etiologies of epilepsy.

Of note, this study was performed as a discovery pilot on a cohort of 20 individuals, and while a number of promising miRNA targets were identified, validation in larger cohorts will be a key next step in evaluating the potential of these targets as individual biomarkers or a combined biomarker signature. The modest sample size limits statistical power for performance estimation and precludes stable discrimination analyses such as ROC curve evaluation, which are prone to overestimation in small discovery cohorts.

While this study provided valuable insight into miRNA-level differences between patients who experienced an IFS and those who went on to develop NOE, our study has several limitations. First, the study was performed in 20 participants. Expanding to a larger cohort could increase statistical power to detect smaller changes in miRNA abundance and provide opportunities for unique sub-group analyses of epilepsy presentations across variables such as age, sex, or race and ethnicity. In addition, analyzing a cohort of patients who had not experienced an FUS could provide valuable insight into potential biomarkers that can be used to determine if a person is more susceptible to seizures. A 2-year longitudinal study of samples taken from the same patient starting at their FUS could also help identify miRNAs that change expression following first seizure and prior to a second seizure. These sorts of data could provide insight into mechanisms of epileptogenic that could be leveraged to devise strategies to prevent or delay the onset of epilepsy.

One significant limitation of this study is the absence of a healthy control group. Consequently, these findings are limited to comparisons within the IFS and NOE groups and do not establish how the identified miRNA signatures differ from healthy individuals. Future studies should include normal controls. In addition, circulating miRNA levels may be influenced by demographic and clinical factors including age, sex, race and ethnicity, medication exposure, comorbidities and timing of blood collection relative to the seizure event. Although efforts were made to account for these relevant covariates, the modest sample size and dual-center nature of this study limited our ability to fully evaluate and adjust for potential confounding factors.

One in ten people are estimated to experience a seizure at some point, and 43%–61% of those individuals have an additional seizure within 2-years. There is a need for an improved, non-invasive method to determine if a patient who suffered a FUS will later be diagnosed with NOE. Existing methods are both ineffective and invasive, and many experts see the utility of a non-invasive biomarker (Enright et al., 2018; Cava et al., 2018; Martinez and Peplow, 2023). Despite this, there are challenges associated with utilizing miRNA as a biomarker for NOE (microRNA, 2025), and additional studies are needed to assess the utility of miRNA as a predictive biomarker. Future studies utilizing miRNA could shed light on potential biomarkers predictive of epilepsy or other neurological disorders. In the future, we intend to build upon this initial analysis in a larger cohort of patients with ISF, NOE, and patients who have not experienced a seizure. A longitudinal analysis with additional blood draws after a patient experienced a FUS could also provide more information about miRNAs that could predict different presentations of epilepsy.

## 5 Conclusion

In summary, we identified distinct circulating miRNA signatures that distinguish patients who present with an ISF from those who develop NOE. These findings provide further evidence that peripheral miRNA profiles may reflect biological processes associated with epileptogensis. However, given the small sample size, and absence of healthy controls, the identified miRNAs should be considered candidate biomarkers rather than clinically actionable markers. Validation in larger, prospective, multi-center studies will be necessary to confirm their reproducibility, determine their diagnostic and prognostic utility, and establish their potential role in clinical decision making.

## Data Availability

The data presented in the study are deposited in the Figshare repository, DOI: 10.6084/m9.figshare.34625283. Deidentified NanoString nCounter RCC files, sample metadata, and count matrices are publicly available at https://doi.org/10.6084/m9.figshare.34625283.
