# Supplementary material for: Exploratory analysis of cell-free miRNA expression changes in patients with isolated first seizure and new-onset epilepsy
Source: Front Mol Biosci. 2026 Oct 30;13:1788160. doi: 10.3389/fmolb.2026.1788160 (PMC13759377; doi:10.3389/fmolb.2026.1788160)
Supplement: Supplementary file 1 [file DataSheet1.pdf]

Supplemental figure S1–Expression of housekeeping genes

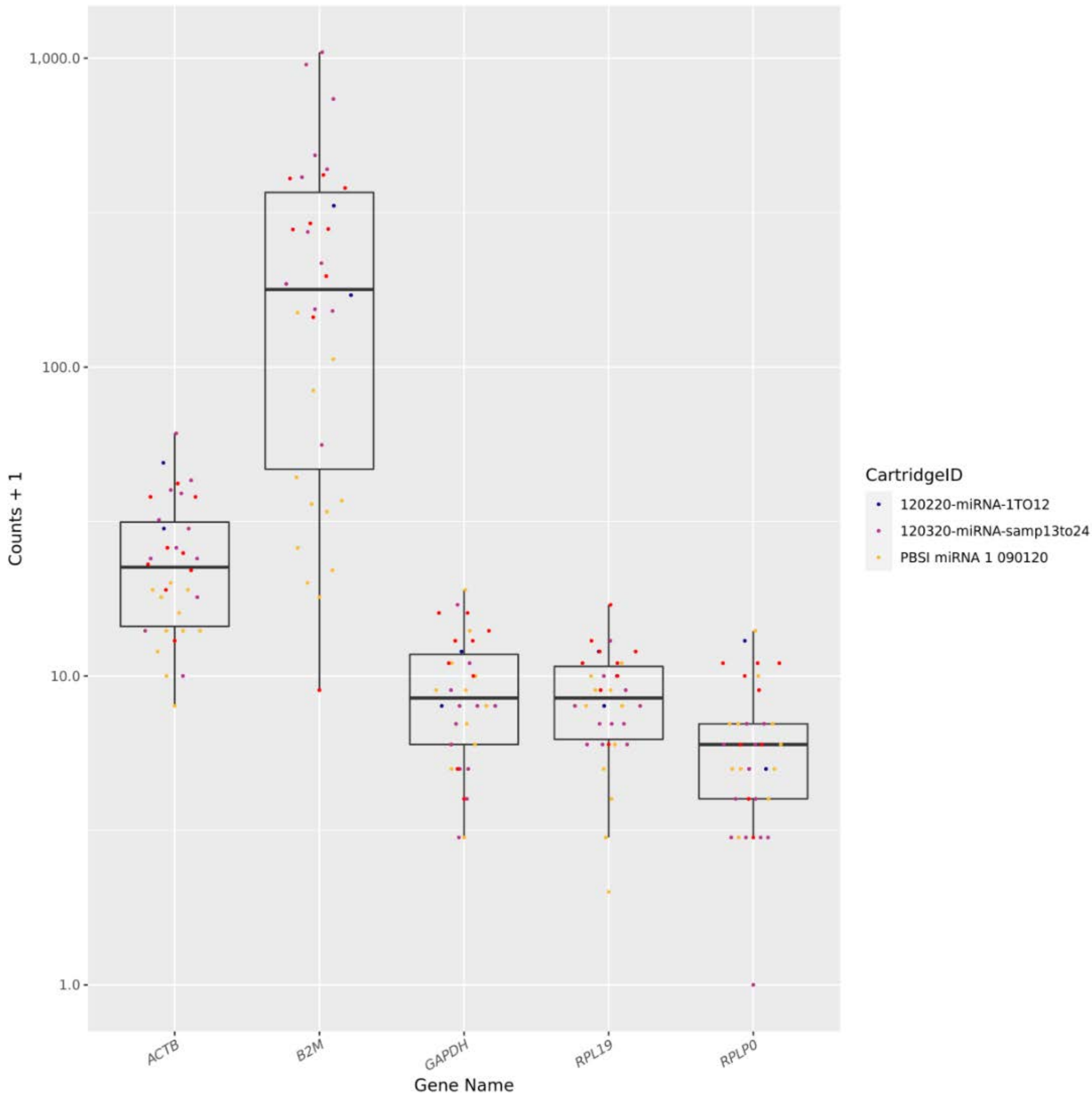

Non-normalized expression of designated housekeeping genes (in counts) used for sample-to-sample normalization.

Supplemental Figure S2–relative log expression plots

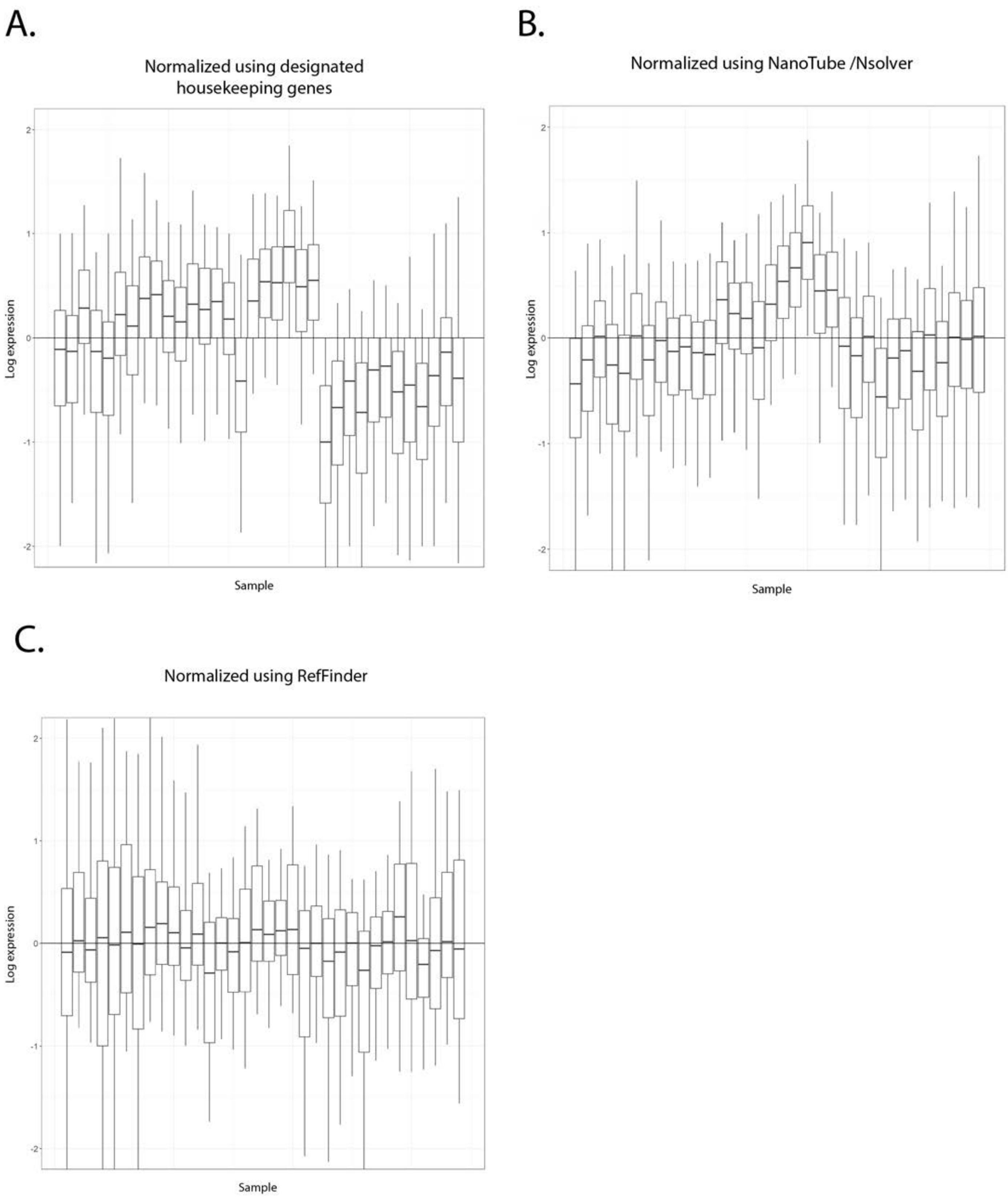

A) RLE plot of sample normalization using designated housekeeping genes. B) RLE plot of sample normalization using NanoTube/Nsolver. C) RLE plot of sample normalization using RefFinder.

Supplemental Table S3–differentially expressed miRNAs

miRNA	log2FoldChange	Q-Value
hsa-miR-579-3p	57.830	0.000
hsa-miR-122-5p	-7.509	0.002
hsa-miR-570-3p	3.324	0.002
hsa-miR-4455	3.497	0.003
hsa-miR-4536-5p	2.627	0.015
hsa-miR-1253	2.071	0.032
hsa-miR-548aa + hsa-miR-548t-3p	2.412	0.032
hsa-miR-548q	2.432	0.032
hsa-miR-548g-3p	2.185	0.038
hsa-miR-502-5p	2.281	0.038
hsa-miR-888-5p	2.228	0.038
hsa-miR-136-5p	-21.812	0.038
hsa-miR-30e-5p	1.856	0.038
hsa-miR-644a	2.171	0.044
hsa-miR-411-5p	2.189	0.045

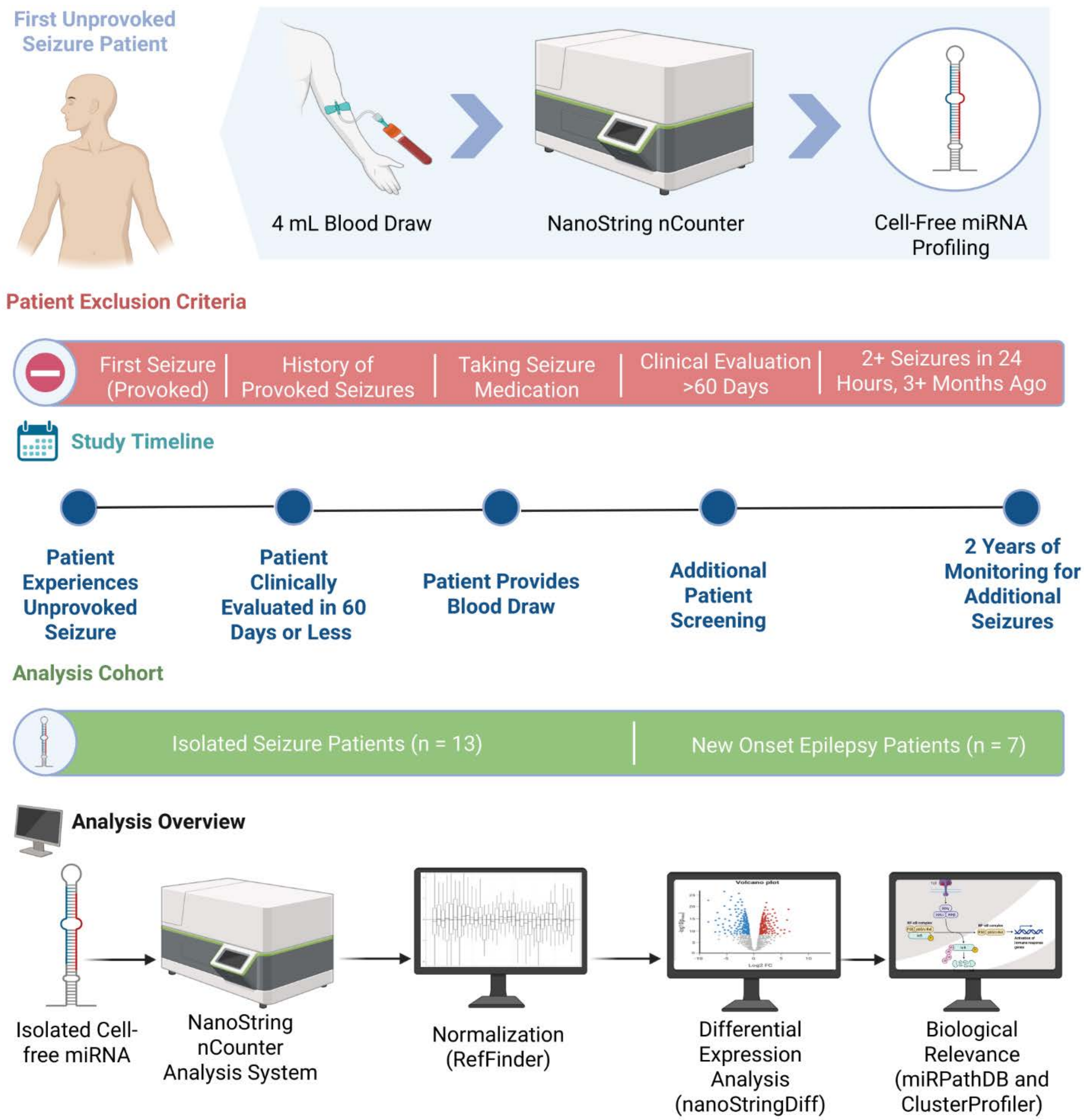

Supplementary Figure S4: Study Overview. Overview of the study procedure, analysis pipeline, reasons for patient exclusion in the study, the timeline of the study following patient consent, and the analysis cohorts. Created in BioRender. Meng, R. (2025) https://BioRender.com/0whm451
